# Macular perfusion normative data acquired with optical coherence tomography angiography in healthy four-year-old Caucasian children

**DOI:** 10.1186/s12886-021-02122-y

**Published:** 2021-10-05

**Authors:** Dunja Bajtl, Mirjana Bjeloš, Mladen Bušić, Ana Križanović, Leon Marković, Biljana Kuzmanović Elabjer

**Affiliations:** 1 University Eye Department, University Hospital Centre Osijek, Osijek, Croatia; 2 Faculty of Medicine, Josip Juraj Strossmayer University of Osijek, Osijek, Croatia; 3 University Eye Department, University Hospital “Sveti Duh”, Sveti Duh 64, 10 000 Zagreb, Croatia; 4 Faculty of Dental Medicine and Health Osijek, Josip Juraj Strossmayer University of Osijek, Osijek, Croatia

**Keywords:** Children, Retina, Perfusion, Angiography, Retinal vessels

## Abstract

**Background:**

The purpose of this cross-sectional study involving healthy emmetropic four-year-old Caucasian children was to provide a macular perfusion normative database acquired with optical coherence tomography angiography (OCTA). One eye of each examinee underwent OCTA imaging. The following parameters were analyzed using AngioTool Image J software: vessels area (VA), vessels density (VD), total number of junctions (TNJ), junctions density (JD), total vessel length (TVL), average vessel length (AVL), total number of endpoints (TNEP), lacunarity (L), vessel diameter index (VDI), tortuosity (T) and foveal avascular zone (FAZ). Average central macular thickness (CMT) and average central macular volume (CMV) were measured.

**Result:**

Sixty-two eyes of 62 children of average age 50.4 ± 3.8 months were examined. VA, VD, and T increased from the inner towards the outer layers of the retina. The intermediate capillary plexus had the highest JD and TNEP and narrowest FAZ. Retinal sexual differentiation was supported with higher values of the retinal VA, VDI and TNEP, and chorioretinal VA, VDI and L in males. The choriocapillaris presented with the highest VD, AVL, and T and the lowest L and TNEP.

**Conclusion:**

The study provides the first detailed normative database of the macular vascular network in the youngest uniform cohort of emmetropic four-year-old children.

**Supplementary Information:**

The online version contains supplementary material available at 10.1186/s12886-021-02122-y.

## Background

Optical coherence tomography angiography (OCTA) provides in vivo imaging of total retinal perfused microvascular networks as well as the choriocapillaris (CC) and choroid displayed as two-dimensional *en face* images derived from 3D volumetric data.

OCTA segmentation of four vascular plexuses agrees with the vascular distribution obtained by histological analysis. The innermost capillary network, the radial peripapillary capillary plexus (RPCP), is found in the nerve fiber layer. The superficial vascular plexus (SVP) is located within the retinal ganglion cell layer and a superficial portion of the inner plexiform layer (IPL). These two layers are further categorized under the OCTA nomenclature as superficial vascular complex (SVC). The intermediate capillary plexus (ICP) is segmented between the deep portion of IPL and the superficial portion of the inner nuclear layer (INL). The outermost layer, the deep capillary plexus (DCP) is established at the level of the deep portion of INL. The latter two capillary networks form a deep vascular complex (DVC).

Extensive cytoarchitectonic studies in vivo are thus feasible enabling important information on the intermediate and deep retinal vascular system that fluorescein angiography (FA) and indocyanine green (ICG) imaging cannot provide. The OCTA has amplified the analysis of microvasculature of CC as dye-based imaging techniques have poor lateral resolution. Data on the macular vascular network in children are limited [1–3], and knowledge on the development of human fovea is mostly gathered via histological studies. As a non-contact, non-invasive tool it can be implemented in cooperative children, in particular when frequent monitoring is needed.

Thus, we performed an analysis of macular microvascular architecture and ocular biometry in children at a one-time point to expand and comprehend the knowledge on macular vascular developmental trajectory during the process of foveal maturation and eye emmetropization.

## Methods

### Study description and oversight

This cross-sectional study was conducted at the University Eye Department, University Hospital “Sveti Duh”, Zagreb, Croatia, between January 2019 and March 2020.

### Study participants

Healthy participants of the Croatian Preventive Program for Early Amblyopia Detection in four-year-old children were recruited in the study [4].

Gender, date of birth, pregnancy, and developmental data for any systemic and eye diseases were obtained from legal guardians. All examinees were tested for visual acuity without correction at near (40 cm) and distance (3 m) using Lea symbols® inline test, binocularly and monocularly adhering to Zagreb Amblyopia Preschool Screening protocol [5]. One eye randomly chosen per examinee underwent OCTA imaging using SPECTRALIS®OCT Angiography (HRA + OCT Spectralis, Heidelberg Engineering, Heidelberg, Germany) followed by axial length measurements acquired with IOLMaster® 700 (Carl Zeiss Meditec AG, Jena, Germany). All measurements were performed by a single experienced operator (D. B.). The imaging was performed in a dark room, with the other eye patched.

Inclusion criteria were defined as follows: (1) visual acuity without correction for distance and for near tested binocularly and monocularly ≤0.1 logMar, (2) OCTA quality index Q ≥ 30.

Exclusion criteria were: (1) presence of any known ocular and/or systemic diseases, malformations, or previous intraocular surgery; (2) inadequate fixation on IOLMaster visible on cross-section scan.

### OCTA imaging

OCTA imaging was performed using an A-scan pattern of 10 × 10° (~ 2.9 × 2.9 mm) containing 512 A-scans × 512 B-scans. A high-resolution scan of 5.7 μm/pixel was applied.

Vascular layers were segmented automatically: RPCP, SVP, ICP, DCP, CC, and choroid. *Retina* slab represented the image of all retinal vessels and *Full* referred to the image of all chorioretinal vessels.

**Fig. 1:**
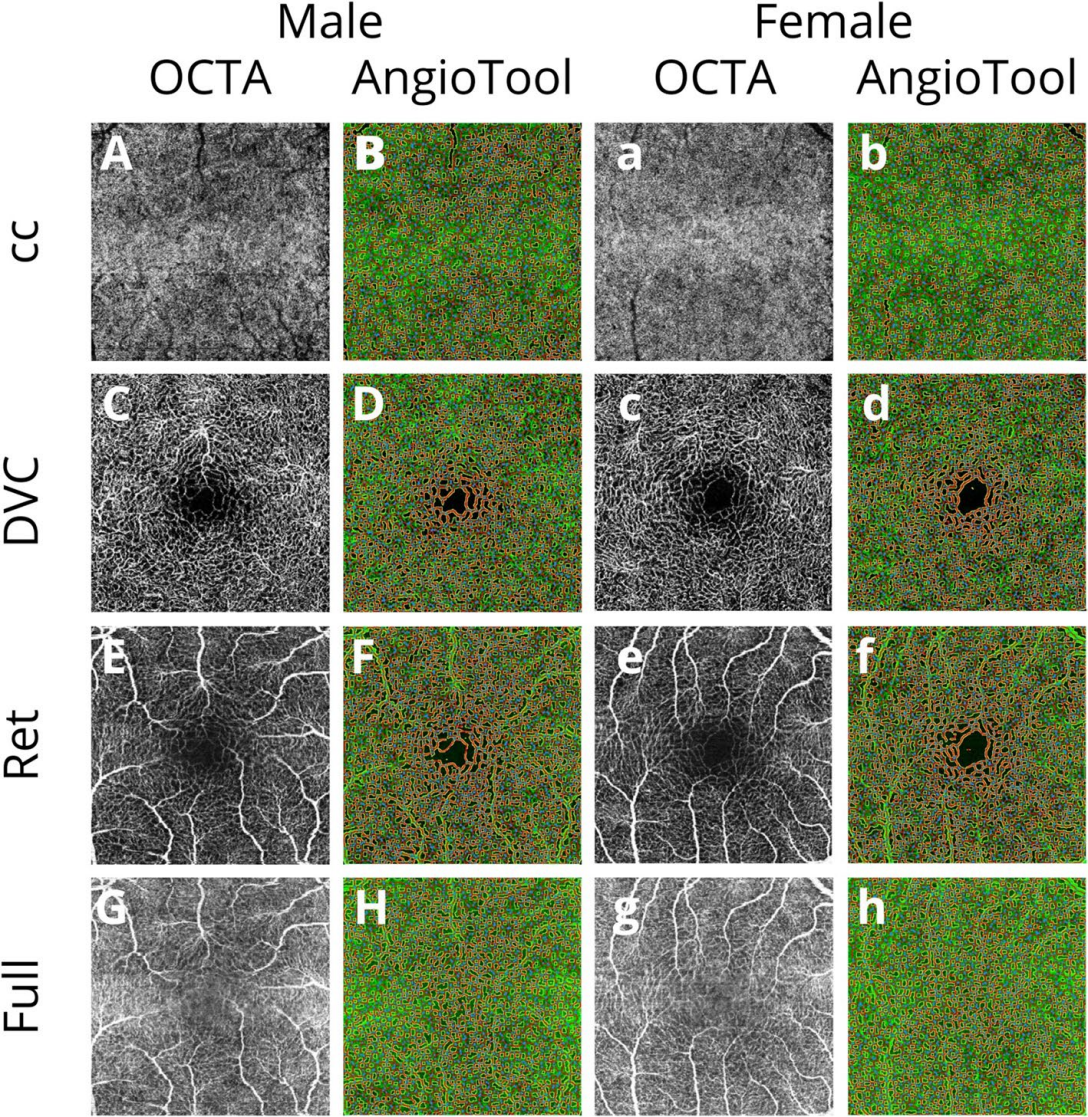
OCTA *en-face* images (image quality Q = 43) showing binary skeletal structure in the left column **A**, **C**, **E**, **G**, **a**, **c**, **e**, **g**, and skeletonized images with overlay of the AngioTool output in the corresponding right column **B**, **D**, **F**, **H**, **b**, **d**, **f**, **h**, of a male (left column) and a female examinee (right column). The skeletons are outlined in red, vessels are presented yellow, branching points are indicated in blue. Calculated vascular parameters are presented in the Table 1

Skeletonized images with Q ≥ 30 dB were further calibrated using 162 pixels per 1 mm to analyze vascular parameters in ~ 8.4 mm^2^ of explanted area (EA) using AngioTool Image J software (version 0.6a, 02.18.14) (Fig. 1). AngioTool is an open-source software provided by the National Cancer Institute (National Institutes of Health®, Bethesda, MD, USA) for vessel parameters analysis: vessels area (area of the segmented vessels inside the explant area, VA), vessels density (percentage of the area of the segmented vessels inside the explant area, VD), total number of junctions (total number of vessel junctions inside the explant area, TNJ), junctions density (number of vessel junctions normalized per unit area, JD), total vessel length (sum of Euclidean distances between the pixels of all the vessels inside the explant area, TVL), average vessel length (mean length of all the vessels inside the explant area, AVL), total number of endpoints (number of open-ended vessel segments, TNEP) and lacunarity (mean lacunarity overall size boxes, L) [6]. A vessel is defined as a segment between a branching point and an endpoint or two branching points [6].

Integrated TruTrack@ Active Eye Tracking corrected displacements of retinal layers due to the reacquisition of images at the correct retinal location, motion, and blinking artifacts [7].

Average central macular thickness (CMT), average central macular volume (CMV), foveolar thickness (FoT), average parafoveolar temporal macular thickness (PTT), average parafoveolar temporal macular volume (PTV), average parafoveolar nasal macular thickness (PNT), average parafoveolar nasal macular volume (PNV), average parafoveolar superior macular thickness (PST), average parafoveolar superior macular volume (PSV), average parafoveolar inferior macular thickness (PIT) and average parafoveolar inferior macular volume (PIV) were obtained automatically using integrated macular thickness map mode.

Foveal avascular zone (FAZ) delineation was performed by a single experienced operator (D. B.) as presented by Zhao et al., for each vascular layer separately, using Adobe Photoshop CC (version 2019 20.0) [8]. Mathematical delineation of FAZ area in mm^2^ is presented in the equation:

$$FAZ=\frac{NP}{ NP M\times M}$$

where *NP* corresponds to the number of pixels enclosed by FAZ area, *NPM* to the number of pixels per mm^2^, and *M* refers to the magnification.

Vessel diameter index (VDI) [9], and tortuosity (T) were calculated according to the following formulas:

$$VDI=1000x\frac{VA}{TVL}$$

$$T=\frac{\left( TNJ+ TNEP-1\right)\times AVL}{TVL}$$

### Biometric eye components measurement

All examinees underwent analysis of the axial length (AL), anterior chamber depth (ACD), lens thickness (LT), central corneal thickness (CCT), corneal curvature in the flattest (K1) and steepest (K2) meridian, horizontal white-to-white (WTW) utilizing IOLMaster® 700 biometer.

The power of the Acrysof IQ Monofocal SN60WF (Alcon Laboratories Inc., Fort Worth, Texas, USA), with A-constant 118.7 was calculated using the SRK-T formula [10].

### Statistical analysis

The primary study outcome was the analysis of vessel parameters VA, VD, TNJ, JD, TVL, AVL, TNEP, L, VDI, and T and metrics on the macular thickness and volume CMT, CMV, FoT, PTT, PTV, PNT, PNV, PST, PSV, PIT, and PIV. Secondary outcomes included assessments of biometric eye components AL, ACD, LT, CCT, K1, K2, WTW, and intraocular lens power.

Categorical data were presented with absolute and relative frequencies, while numerical data were presented with arithmetic mean and standard deviation if distributed within parameters of normal Gaussian distribution. Numerical data that did not fit the normal distribution were presented with median and interquartile range.

Differences between the two independent sets of numerical data were tested with the nonparametric Mann-Whitney U test, while differences between more than two dependent sets of numerical data were tested with the nonparametric Friedman test and Conover post-hoc test. The sexual differentiation for each of the parameters of primary and secondary outcomes. Statistical analysis was performed with MedCalc (19.1.3, MedCalc Software bv) and IBM SPSS Statistics (release 24.0.0.0) software tools, with statistical significance defined as α = 0.05, where all *P* values were two-tailed.

## Results

### Study participants

The study examined 62 eyes of 62 children of average age 50.4 ± 3.8 (mean ± SD) months (range 48 to 59 months of age). There were 30 (48.4%) right eyes, and 32 (51.6%) left (χ^2^ test, *P* = 0.86). Twenty-nine (46.8%) children were male and thirty-three (53.2%) were female (χ^2^ test, *P* = 0.72). EA was equal across all vascular layers. Statistical difference was not found (Friedman test; *P* = 0.12).

### Primary outcome measures

Table 1 presents normative data of sexual differentiation of examined vascular parameters VA, VD, TNJ, JD, TVL, AVL, TNEP, L, VDI, T, FAZ area, macular thickness, and volume (Mann-Whitney U test, *P* < 0.05). The distribution of the vascular parameters VA, VD, TNJ, JD, TVL, AVL, TNEP, L, VDI, T, and FAZ area across the layers is presented in Additional file 1: Table S1. The macular thickness and volume distribution are presented in Additional file 1: Table S2. Heterogeneity of vascular layers for the vascular parameters VA, VD, TNJ, JD, TVL, AVL, TNEP, L, VDI, T, and FAZ area is analyzed in Additional file 1: Table S3 (Conover post-hoc test, *P* < 0.05).

**Table 1:** Sexual differentiation of examined vascular parameters

Variable (measurement unit)	Layer	Male			Female			*P* Value^*^
		Mean	25th percentile	75th percentile	Mean	25th percentile	75th percentile	
EA (mm^2^)	RPCP	8.38	8.38	8.38	8.38	8.38	8.38	0.64
	SVP	8.38	8.38	8.38	8.38	8.38	8.38	0.16
	ICP	8.38	8.38	8.38	8.38	8.38	8.38	0.62
	DCP	8.38	8.38	8.38	8.38	8.38	8.38	0.08
	CC	8.38	8.38	8.38	8.38	8.38	8.38	0.09
	Choroid	8.38	8.38	8.38	8.38	8.38	8.38	0.40
	SVC	8.38	8.38	8.38	8.38	8.38	8.38	0.07
	DVC	8.38	8.38	8.38	8.38	8.38	8.38	0.23
	Ret	8.38	8.38	8.38	8.38	8.38	8.38	0.56
	Full	8.38	8.38	8.38	8.38	8.38	8.38	0.62
VA (mm^2^)	RPCP	3.44	2.89	3.94	3.32	2.64	3.65	0.38
	SVP	5.46	5.27	5.69	5.43	5.08	5.61	0.30
	ICP	5.53	5.37	5.63	5.45	5.17	5.60	0.31
	DCP	5.70	5.55	5.85	5.60	5.45	5.78	0.16
	CC	6.69	6.45	6.80	6.61	6.50	6.78	0.97
	Choroid	5.46	5.29	5.54	5.57	5.38	5.72	0.05
	SVC	5.04	4.87	5.33	4.97	4.52	5.20	0.15
	DVC	5.93	5.78	6.09	5.81	5.62	6.02	0.08
	Ret	5.43	5.14	5.62	5.20	4.94	5.49	**0.03**
	Full	5.72	5.53	5.78	5.51	5.37	5.74	**0.04**
VD (%)	RPCP	41.07	34.54	46.98	39.64	31.47	43.55	0.38
	SVP	65.12	62.91	67.91	64.84	60.59	66.95	0.31
	ICP	66.02	64.08	67.19	64.98	61.72	66.87	0.31
	DCP	68.02	66.20	69.84	66.81	65.10	69.03	0.16
	CC	79.86	76.98	81.13	78.87	77.61	80.88	0.97
	Choroid	65.17	63.09	66.09	66.53	64.27	68.21	0.05
	SVC	60.14	58.14	63.56	59.29	53.97	62.12	0.16
	DVC	70.76	68.93	72.67	69.38	67.06	71.87	0.08
	Ret	64.82	61.37	67.10	62.04	59.01	65.52	**0.03**
	Full	68.32	66.04	68.95	65.76	64.13	68.48	**0.04**
TNJ	RPCP	1196.00	948.50	1391.50	1114.00	831.50	1235.00	0.23
	SVP	1530.00	1486.50	1581.50	1516.00	1471.50	1564.00	0.33
	ICP	1763.00	1715.00	1802.50	1757.00	1695.50	1789.00	0.45
	DCP	1696.00	1653.50	1737.50	1692.00	1636.50	1737.00	0.69
	CC	1628.00	1596.50	1721.00	1658.00	1610.50	1742.00	0.23
	Choroid	1625.00	1594.00	1655.50	1602.00	1551.50	1674.00	0.59
	SVC	1494.00	1432.50	1589.50	1496.00	1381.00	1573.00	0.37
	DVC	1781.00	1733.00	1834.50	1762.00	1704.50	1807.00	0.11
	Ret	1781.00	1743.50	1858.00	1781.00	1699.50	1813.50	0.20
	Full	1910.00	1868.00	1930.50	1910.00	1875.50	1930.50	0.75
JD (mm^2^)	RPCP	142.73	113.20	166.04	132.95	99.22	147.37	0.23
	SVP	182.56	177.39	188.73	180.91	175.57	186.65	0.33
	ICP	210.38	204.65	215.09	209.68	202.33	213.47	0.46
	DCP	202.38	197.31	207.33	201.90	195.28	207.27	0.70
	CC	194.28	190.50	205.34	197.82	192.19	207.86	0.23
	Choroid	193.88	190.21	197.54	191.15	185.15	199.76	0.59
	SVC	178.31	170.97	189.70	178.49	164.78	187.71	0.39
	DVC	212.51	206.80	218.89	210.25	203.43	215.62	0.11
	Ret	212.60	208.03	221.72	212.51	202.81	216.42	0.20
	Full	227.89	222.92	230.37	227.95	223.78	230.39	0.76
TVL (mm)	RPCP	125.30	105.90	137.76	117.72	97.55	128.03	0.22
	SVP	152.15	148.52	154.93	151.45	148.23	153.68	0.31
	ICP	163.42	161.20	164.87	163.64	159.22	164.91	0.64
	DCP	159.53	156.21	161.72	159.00	155.86	161.49	0.48
	CC	159.86	158.32	163.56	162.00	159.23	164.14	0.29
	Choroid	157.13	154.26	158.96	156.45	153.39	159.96	0.91
	SVC	149.66	146.34	154.77	149.39	141.86	152.77	0.35
	DVC	164.29	161.98	166.37	163.34	161.11	165.04	0.10
	Ret	164.14	161.20	167.02	163.44	159.66	165.66	0.14
	Full	170.18	168.52	171.23	169.68	166.94	170.94	0.29
AVL (mm)	RPCP	0.73	0.40	1.01	0.56	0.30	0.76	0.17
	SVP	8.71	7.07	13.50	7.87	5.46	10.85	0.13
	ICP	10.11	7.04	16.57	9.11	7.09	13.54	0.54
	DCP	11.14	9.75	14.53	11.46	9.89	14.40	0.85
	CC	55.44	39.86	82.59	78.95	46.70	120.32	0.57
	Choroid	11.89	7.76	21.15	15.42	9.48	26.76	0.18
	SVC	7.57	4.60	9.56	6.36	3.89	7.99	0.11
	DVC	27.87	20.50	40.62	20.10	11.57	32.92	**0.01**
	Ret	7.20	5.21	12.51	5.89	4.70	9.14	0.13
	Full	28.09	16.19	49.45	18.63	13.44	31.18	**0.04**
TNEP	RPCP	734.00	598.50	878.00	778.00	655.50	931.00	0.36
	SVP	143.00	124.00	180.00	154.00	134.50	196.00	0.13
	ICP	148.00	131.50	179.00	162.00	126.50	199.00	0.41
	DCP	128.00	106.50	145.00	131.00	111.50	151.50	0.47
	CC	59.00	53.00	75.00	64.00	55.00	74.50	0.86
	Choroid	206.00	174.50	236.50	181.00	152.50	222.00	0.08
	SVC	180.00	149.50	237.50	196.00	171.00	304.00	0.08
	DVC	93.00	77.50	105.00	93.00	81.50	129.00	0.22
	Ret	171.00	144.00	219.00	204.00	165.50	276.00	**0.03**
	Full	133.00	109.50	167.00	162.00	126.50	198.50	0.07
L^**^	RPCP	0.11	0.08	0.14	0.14	0.09	0.19	0.05
	SVP	0.03	0.02	0.04	0.03	0.03	0.04	0.46
	ICP	0.02	0.01	0.02	0.02	0.02	0.03	0.62
	DCP	0.03	0.03	0.03	0.03	0.03	0.04	0.45
	CC	0.01	0.01	0.01	0.01	0.01	0.01	0.98
	Choroid	0.01	0.01	0.01	0.01	0.01	0.01	0.18
	SVC	0.04	0.03	0.05	0.04	0.04	0.05	0.21
	DVC	0.02	0.02	0.02	0.02	0.02	0.03	0.19
	Ret	0.02	0.02	0.02	0.02	0.02	0.03	0.10
	Full	0.0107	0.0093	0.0126	0.0122	0.0111	0.0132	**0.026**
FAZ area (mm^2^)	RPCP	1.13	0.72	1.42	1.27	0.93	1.61	0.10
	SVP	0.55	0.36	0.67	0.52	0.41	0.64	0.99
	ICP	0.22	0.13	0.29	0.23	0.12	0.32	0.76
	DCP	0.52	0.43	0.59	0.53	0.38	0.65	0.64
	SVC	0.62	0.44	0.78	0.65	0.45	0.75	0.86
	DVC	0.26	0.15	0.35	0.30	0.17	0.36	0.48
	Ret	0.28	0.17	0.33	0.31	0.17	0.39	0.36
VDI (μm)	RPCP	27.62	26.90	28.72	27.74	26.37	29.05	0.84
	SVP	35.90	34.56	36.95	35.68	34.03	36.57	0.41
	ICP	33.66	33.00	34.22	33.28	32.62	34.15	0.31
	DCP	35.66	34.44	37.33	35.21	34.18	36.19	0.27
	CC	41.99	39.20	42.82	41.50	39.63	42.28	0.79
	Choroid	34.60	33.69	35.44	35.62	34.43	36.05	**0.01**
	SVC	33.75	32.72	34.61	33.08	31.60	34.23	0.14
	DVC	35.91	35.01	37.48	35.39	34.66	36.67	0.19
	Ret	32.68	31.13	34.22	31.82	31.09	33.02	0.05
	Full	33.42	32.82	33.99	32.47	31.91	33.66	**0.04**
T	RPCP	11.14	6.82	14.84	8.98	5.57	11.54	0.15
	SVP	96.56	78.10	148.27	85.68	60.94	119.11	0.11
	ICP	114.13	83.46	193.42	106.17	83.25	157.75	0.49
	DCP	128.86	111.69	168.55	131.57	113.74	163.36	0.92
	CC	612.67	421.25	925.50	835.50	499.96	1251.00	0.48
	Choroid	139.77	90.53	240.73	173.00	108.25	309.33	0.20
	SVC	80.90	53.69	105.72	71.50	44.88	89.21	0.15
	DVC	323.50	232.81	453.88	224.75	129.56	365.90	**0.01**
	Ret	87.26	63.10	148.11	71.36	58.17	110.36	0.15
	Full	341.67	196.35	568.79	229.67	161.56	370.33	**0.04**
Macular thickness (μm)	CMT	252.00	239.00	267.50	256.00	240.50	265.00	0.76
	FoT	204.00	197.50	213.00	207.00	199.50	216.50	0.32
	PTT	328.00	314.00	334.50	324.00	319.00	333.00	0.89
	PNT	335.00	324.50	347.00	336.00	327.00	344.50	0.78
	PST	338.00	326.50	348.00	339.00	332.50	347.50	0.53
	PIT	339.00	325.50	344.00	334.00	327.50	343.00	0.52
Macular volume (μl)	CMV	0.20	0.19	0.21	0.20	0.19	0.21	0.94
	PTV	0.52	0.49	0.52	0.51	0.50	0.52	0.69
	PNV	0.53	0.51	0.54	0.53	0.51	0.54	0.71
	PSV	0.53	0.51	0.55	0.53	0.52	0.55	0.46
	PIV	0.53	0.51	0.54	0.53	0.51	0.54	0.66

*EA* explant area, *VA* vessels area, *VD* vessels density, *TNJ* total number of junctions, *JD* junctions density, *TVL* total vessel length, *AVL* average vessel length, *TNEP* total number of endpoints, *L* mean lacunarity, *FAZ* foveal avascular zone, *VDI* vessel diameter index, *T* tortuosity, *RPCP* radial peripapillary capillary plexus, *SVP* superficial vascular plexus, *ICP* intermediate vascular plexus, *DCP* deep capillary plexus, *SVC* superficial vascular complex, *DVC* deep vascular complex, *CC* choriocapillaris, *Ret* retina, *Full* chorioretina, *CMT* central macular thickness, *CMV* central macular volume, *FoT* foveolar thickness, *PTT* parafoveal temporal thickness, *PTV* parafoveal temporal volume, *PNT* parafoveal nasal thickness, *PNV* parafoveal nasal volume, *PST* parafoveal superior thickness, *PSV* parafoveal superior volume, *PIT* parafoveal inferior thickness, *PIV* parafoveal inferior volume

*Statistical significance was measured using Mann-Whitney U Test. Bolded values denote statistical significance at the level *P* < 0.05

**The chorioretinal lacunarity value is presented with four decimal places due to better observation of statistically significant differences

### Secondary outcome measures

The analyzed biometric eye components AL, ACD, LT, CCT, K1, K2, WTW, and refractive eye power (Student’s t-test, *P* < 0.05) (Table 2) did not demonstrate sexual dimorphism in four-year-old Caucasian children (Additional file 1: Table S4).

**Table 2:** Globe biometry normative values with gender distribution in four-year-old Caucasian children (*n* = 62)

Component (measurement unit)	Male (*n* = 29)		Female (*n* = 33)		*P* value^*^
	mean	SD	mean	SD	
Age (months)	50.03	3.81	50.61	3.75	0.56
AL (mm)	22.29	0.56	22.05	0.48	0.08
ACD (mm)	3.44	0.25	3.39	0.29	0.50
LT (mm)	3.67	0.15	3.70	0.23	0.52
SE (D)	43.27	1.29	43.59	1.13	0.31
K1 (D)	42.88	1.25	43.16	1.09	0.35
K2 (D)	43.68	1.36	44.01	1.22	0.31
CCT (μm)	541.48	31.20	537.21	29.53	0.58
WTW (mm)	12.30	0.39	12.28	0.36	0.85
IOL (D)^**^	25.55	1.10	25.94	1.36	0.23

*n* number of participants, *AL* axial length, *ACD* anterior chamber depth, *LT* lens thickness, *K1* flat corneal meridian, *K2* steep corneal meridian, *SE* spherical equivalent, *CCT* central corneal thickness, *WTW* white-to-white, *IOL* intraocular lens, *SD* standard deviation

*Statistical significance was measured using Student’s t-test for independent samples *(P* < 0.05)

**Acrysoft IQ monofocal SN60WF (Alcon Laboratories Inc., Fort Worth, Texas, USA), A-constant 118.7 using SRK-T formula

## Discussion

This study provides the first detailed quantitative delineation of the macular vascular network in the youngest uniform cohort of emmetropic four-year-old children of Caucasian origin.

Uniform Explant area (Friedman test; *P* = 0.12) across layers enabled reliable further statistical analysis of the measured primary endpoints.

The morphological development of the macula takes place predominantly during the first 5 years of life, thus demonstrating different timeframes of layers development. The development of the inner retinal layers is completed by 5 months [11], while adult human cone density is reached between 4 and 6 years of age [12]. A full adult architecture is reached by 10 years of age [9]. This is the first study revealing that retinal VA, VD, and T increased from the inner towards the outer layers of the retina with SVP and ICP demonstrating no difference in VA and VD (Additional file 1: Tables S1, S3). This is consistent with the functional distribution of the regions of the highest oxygen demands and light absorption as follows: inner plexiform layer, outer plexiform layer, and photoreceptor layer.

On the contrary, in adult human donor eye, capillary density was the highest in networks supplying the IPL, namely SVP and ICP, and lowest in DVP [13]. Thus, it is possible that the microvascular model observed in this study extends until the normal aging process commences. The characteristic pattern of the normal aging affecting adults after 35 years of age is presented with decreased retinal tissue perfusion in DVP as the primary event. This is followed by further changes in volumetric vessel density in DVP and SVP in opposite directions, demonstrating a decrease and increase, respectively [14].

We found significantly larger vessel areas in boys in the DVC, retina layer and the entire chorioretina compared to girls (Table 1). This poses an important question: whether boys mature earlier and similarity between genders evolves later in the developmental process? The alternative is that this structural dimension of sexual dimorphism is finite and plays a protective role during aging as macular perfusion decreases more rapidly in males [9]. The latter scenario could be reinforced with observed variations in the foveal capillary-free area being larger in healthy adult females [1, 15–17].

Retinal sexual differentiation was further supported with higher values of the retinal TNEP and chorioretinal lacunarity in males compared to their female counterparts (Table 1). Lacunarity discloses a more heterogeneous spatial arrangement of the vessels in males. We speculate if this component could be the coupled to complement the process of a more rapid decline of perfusion in males [15].

Sex-based differences in neuroretinal function presume a causal relationship to estradiol (17ß-estradiol, E2) levels [18]. Increased retinal thickness in male rat retina is influenced by prenatal and especially neonatal testosterone surge, not evidenced in females, and its local conversion to estradiol [19]. E2 induces angiogenesis by enhancing the VEGF expression, vasodilation, and thus increased blood flow [20]. The inner nuclear layer, the only layer supported by two capillary networks, ICP and DCP, is reported to be the main site of E2 synthesis [21]. Furthermore, INL neurons control photoreceptor homeostasis by sustaining structural and trophic support to the vasculature [22].

The CC demonstrated the highest vessel area, vessel density, average vessel length, vessel diameter index and tortuosity with the lowest lacunarity and the total number of endpoints. This is closely related to the high metabolic burden of photoreceptors and the key role of choroidal circulation in delivering nutrients and oxygen to the outer retinal layers, as well as in keeping the steady temperature. In healthy individuals, swept-source optical coherence tomography angiography (SS-OCTA) of the vessel density is within the range of the morphometric results assessed with tissue microscopy [22]. During normal aging (range 6–100 years) the CC vessel density and capillary diameter decrease linearly from 0.75 and 9.8 μm in the first decade to 0.41 and 6.5 μm in the tenth decade, respectively [23]. In four-year-old children, we measured a higher CC vessel density of 79.74% (Additional file 1: Table S1). Sugano et al. observed healthy individuals aged 27.9 ± 5.8 using SS-OCTA and AngioTool software and reported lacunarity of the choriocapillaris 0.016 ± 0.001 [24]. As a comparison, in this study, the CC mean lacunarity was 0.00564 ± 0.01. The highest tortuosity of CC supports the proposed thermoregulatory function of the choroid as coiling increases the surface area to volume ratio resulting in more effective heat dissipation.

To give a more thorough insight into the perifoveal capillary plexus formation, we observed FAZ separated across four vascular retinal slabs (Table 1), although it has been recommended that the FAZ size should be measured using an *en face* projection that includes all retinal plexuses [1], rather than separating the FAZ area across the layers [25]. In our study, the FAZ area varied across capillary plexuses, but these differences were not found to be sex-dependent (Table 1, Additional file 1: Table S1). The FAZ area was the narrowest in ICP (Additional file 1: Table S1), while DCP and SVP indicated no difference in the FAZ size (Additional file 1: Table S3). The explanation for these patterns can be found in different mechanisms governing the development of the foveal capillaries. First, the SVP layer sets the boundary defining the presumed foveal avascular area before the foveal depression begins to form [26]. Opposite to the peripheral retina, at the fovea, DVP forms prior to ICP by downward sprouting of SVP capillaries [27]. This sequence of events may be due to the increased metabolic needs of central photoreceptors [26]. The sprouting capillaries from DVP then ascend into IPL to form ICP. Thus, SVP and DCP anastomose first, in the early postnatal period on the margin of the avascular area, while ICP forms anastomosis later, on the foveal slope. The former could provide the reasoning for similarities in VDI between SVP and DCP (Additional file 1: Table S3). This temporal and spatial pattern of the retinal vasculature growth is related to the total number of blind end capillaries being the lowest at DCP and the highest at ICP (Table 1, Additional file 1: Table S3). Moreover, ICP had the highest JD (Table 1, Additional file 1: Table S3). The JD is a measure of sprouting activity and indicates higher angiogenesis [6]. Provis et al. demonstrated a spatial correspondence between the astrocyte-vascular ring and the foveal rim, postulating that blind-ending capillaries directed into the foveal region indicate the presence of inhibitory vascular factors that generate a “no-go” region at the immature fovea [26]. We speculate that ICP angiogenesis must be extensive as the layer ties in with DCP and SVP, but the process itself could be halted at the same time as for DCP and SVP, leaving the highest TNEP. As the constitution of the foveal depression and perifoveal plexuses development are interdependent events, JD and TNEP analysis at different time points may further clarify the period of the foveal maturation.

Even in adults, data of the FAZ area in ICP are scarce. Park JJ et al. observed that FAZ was qualitatively the smallest and best demarcated at ICP, however, quantitative measurements were not performed [28]. Given our results, it is evidenced that this feature is thus already modelled at the age of four.

From the age of eight onwards, the FAZ area observed at SVP only was smaller compared to our study group [1, 29]. Zhang et al. evaluated sex-related differences and a significantly larger FAZ area was found in girls [1]. Our results of the larger FAZ area indicate that at the age of four, foveal pit is still widening and shallowing due to retinal stretch as a major factor in cone packing [27]. In contrast to retinal stretching, pars plana growth adjusts for further posterior eye elongation afterward [30]. In adults, an annual increase in the FAZ area was reported [28, 31].

For full-term born preschool children, normative data on macular thickness are limited [32], while metrics of the concomitant macular microvascular network are even more scarce [23]. How the components of thickness and vascular network are further coupled has yet to be declared. The central macula was the thinnest (253.82 μm). The macular thickness of superior (338.03 μm), inferior (334.32 μm), and nasal (335.39 μm) parafoveal areas were similar, while temporal parafoveolar area was thinner (324.63 μm). However, the distribution of macular thickness and its sexual differentiation did not reach statistical significance (Table 1, Additional file 1: Table S2). Compared with literature values for six-year-old children, our results could indicate that after the age of four the axial length is further increasing, the inner retina is thinning and foveal depression is still deepening. In our study, the topographic retinal profile of the thinnest temporal and the thickest superior retinal inner segment observed at the age of 4 reached significance at the age of six [32]. Furthermore, sexual dimorphism of the central and inner macula thickness and central macular volume is not recorded before the age of six, with boys reaching a higher value [32]. Ethnic differences in macular thickness demonstrate significantly thicker retinas in East Asian compared to white 6-year-old children [32].

Our results indicate that at the age of 4, ocular components AL and corneal power are within the asymptotic phase of growth (Additional file 1: Table S4). The mean axial length in our study was 22.17 ± 0.53 mm, consistent with earlier reports [33–37]. A slow increase in central corneal thickness and ACD until 10 years of age was observed [35, 37]. Our results, measuring CCT 539.21 ± 30.15 μm compare favorably with earlier studies of similar age groups [36, 38], however, these published data are not within the emmetropic refractive range. In the Gutenberg Eye Study, mean CCT was 557.3 ± 34.3 μm in male and 551.6 ± 35.2 μm in female adult subjects (age range 35–74 years) [39]. Mean ACD and LT in our study were 3.42 ± 0.27 mm and 3.69 ± 0.19 mm, respectively. For this age group, we could not find corresponding values obtained with partial coherence laser interferometry in published literature. Compared to the adult Caucasians’ values for ACD 3.87 ± 0.35 mm [40], LT of 4.05 ± 0.20 mm [41], and WTW 13.23 ± 0.44 [42], our results are largely distinctive (Additional file 1: Table S4). The present study identified that apart from the chorioretina, sex-based differences were not found for any other biometric ocular components. Sex-related differences emerge during school age with shorter axial length [42], shallower ACD [37, 38], and steeper corneal curvatures in girls [43, 44]. However, other studies did not confirm dimorphism [45]. Given our results at the age of four, the eye has a power of 25.76 ± 1.25 D.

Although a relatively large sample size of uniform age was enrolled, we did not perform a longitudinal study to assess temporal trends of macular maturation. Objective refraction was not addressed in this study; therefore, this bias may cause some data deformation, however, a high threshold of ≤0,1 logMAR was set to define normative visual acuity in four-year-old children.

## Conclusions

To the best of our knowledge, this is the first study that measured tortuosity across all layers. Although considered to be a more robust indicator of vascular function compared to vessel width, the nonexistence of a universally adopted mathematical definition of tortuosity precludes its use in large population studies and its relation to local and systemic diseases. We believe that this proposed quantitative vessel tortuosity index can be used in future studies to further define its diagnostic potential as the indicator of early retinal and systemic pathology since it is operator-independent and easy to calculate. Due to the simplicity of AngioTool analysis, morphometric parameters, namely lacunarity, could be used as a noninvasive valuable tool in defining early amblyopia [46].

This database acquired baseline information on physiological vascular parameters among normal healthy emmetropic children needed for assessment of macular microvascular architecture impairments, detection of early inflammation response as well as monitoring response to drug and surgical treatment.

Further studies are needed to reveal temporal trends of macular vascular network maturation and sexual dimorphism.

## Supplementary Information

**Additional file 1: Table S1.** Distribution of the vascular parameters and FAZ surface of different examined areas for all eyes in the study (*n* = 62). **Table S2.** Distribution of macular thickness and volume. **Table S3.** Post-hoc analysis of the examined variables. **Table S4.** Globe biometry normative values in four-year-old Caucasian children (*n* = 62).

## Data Availability

The datasets generated and/or analyzed during the current study are not publicly available due to the extensive and large-scale datasheets, but are available from the corresponding author on reasonable request.

## Abbreviations

ACD: Anterior chamber depth
AL: Axial length
AVL: Average vessel length
CC: Choriocapillaris
CCT: Central corneal thickness
CMT: Central macular thickness
CMV: Central macular volume
DCP: Deep capillary plexus
DVC: Deep vascular complex
E2: 17ß-estradiol
EA: Explant area
FA: Fluorescein angiography
FAZ: Foveal avascular zone
FoT: Foveolar thickness
ICG: Indocyanine green
ICP: Intermediate capillary plexus
INL: Inner nuclear layer
IPL: Inner plexiform layer
JD: Junctions density
K1: Corneal curvature in the flattest meridian
K2: Corneal curvature in the steepest meridian
L: Lacunarity
LT: Lens thickness
M: Magnification
NP: Number of pixels
NPM: Number of pixels per mm2
OCTA: Optical coherence tomography angiography
PCV: Parafoveolar superior macular volume
PIT: Parafoveolar inferior macular thickness
PIV: Parafoveolar inferior macular volume
PNT: Parafoveolar nasal macular thickness
PNV: Average parafoveolar nasal macular volume
PST: Parafoveolar superior macular thickness
PTT: Parafoveolar temporal macular thickness
PTV: Parafoveolar temporal macular volume
RPCP: Radial peripapillary capillary plexus
SS-OCTA: Swept-source optical coherence tomography angiography
SVC: Superficial vascular complex
SVP: Superficial vascular plexus
T: Tortuosity
TNEP: Total number of endpoints
TNJ: Total number of junctions
TVL: Total vessel length
VA: Vessels area
VD: Vessels density
VDI: Vessel diameter index
WTW: White-to-white
