# Supplementary material for: Macular perfusion normative data acquired with optical coherence tomography angiography in healthy four-year-old Caucasian children
Source: BMC Ophthalmol. 2021 Oct 5;21:354. doi: 10.1186/s12886-021-02122-y (PMC8491392; doi:10.1186/s12886-021-02122-y)
Supplement: Supplementary file 1 — Additional file 1: Table S1. Distribution of the vascular parameters and FAZ surface of different examined areas for all eyes in the study (n = 62). Table S2. Distribution of macular thickness and volume. Table S3. Post-hoc analysis of the examined variables. Table S4. Globe biometry normative values in four-year-old Caucasian children (n = 62). [file 12886_2021_2122_MOESM1_ESM.pdf]

Title: Macular perfusion normative data acquired with optical coherence tomography angiography in healthy four-year-old Caucasian children

Authors: Dunja Bajtl, MD,[1] Mirjana Bjeloš, MD, PhD,[2,3,4] Mladen Bušić, MD, PhD,[2,3,4] Ana Križanović, MD,[3,4] Leon Marković, MD,[3,4] Biljana Kuzmanović Elabjer, MD, PhD[2,3,4]

[1] University Eye Department, University Hospital Centre Osijek, Osijek, Croatia

[2] Faculty of Medicine, Josip Juraj Strossmayer University of Osijek, Osijek, Croatia

[3] University Eye Department, University Hospital „Sveti Duh“, Zagreb, Croatia

[4] Faculty of Dental Medicine and Health Osijek, Josip Juraj Strossmayer University of Osijek, Osijek, Croatia

Table S1 Distribution of the vascular parameters and FAZ surface of different examined areas for all eyes in the study (n = 62)

Examined area	Minimum	25th percentile	Median	75th percentile	Maximum
**EA** (mm^2)					
RPCP	8.38	8.38	8.38	8.38	8.38
SVP	8.38	8.38	8.38	8.38	8.38
ICP	8.38	8.38	8.38	8.38	8.38
DCP	8.38	8.38	8.38	8.38	8.38
CC	8.38	8.38	8.38	8.38	8.38
Choroid	8.38	8.38	8.38	8.38	8.38
SVC	8.38	8.38	8.38	8.38	8.38
DVC	8.38	8.38	8.38	8.38	8.38
Ret	8.38	8.38	8.38	8.38	8.38
Full	8.38	8.38	8.38	8.38	8.38
**VA** (mm^2)					
RPCP	1.53	2.56	3.14	3.68	4.70
SVP	4.33	5.18	5.40	5.59	5.81
ICP	4.34	5.24	5.47	5.60	6.10
DCP	5.05	5.49	5.63	5.80	6.39
CC	5.49	6.54	6.68	6.82	7.01
Choroid	4.66	5.23	5.50	5.68	6.66
SVC	3.45	4.67	4.97	5.21	5.42
DVC	4.95	5.67	5.89	6.02	6.48
Ret	4.45	5.02	5.29	5.54	5.92
Full	4.30	5.42	5.62	5.76	6.12
**VD** (%)					
RPCP	18.29	30.56	37.52	43.95	56.04
SVP	51.72	61.78	64.42	66.75	69.27
ICP	51.82	62.50	65.21	66.85	72.77
DCP	60.30	65.49	67.22	69.19	76.21
CC	65.51	78.08	79.74	81.34	83.61
Choroid	55.55	62.39	65.64	67.83	79.42
SVC	41.23	55.77	59.25	62.22	64.71
DVC	59.03	67.62	70.27	71.79	77.32
Ret	53.08	59.86	63.07	66.09	70.67
Full	51.29	64.61	67.04	68.77	72.96

**TNJ (N)**					
RPCP	362.00	828.00	1072.00	1279.00	1587.00
SVP	1211.00	1469.00	1522.00	1564.00	1681.00
ICP	1413.00	1709.00	1751.50	1788.00	1858.00
DCP	1528.00	1645.00	1695.00	1724.00	1855.00
CC	1527.00	1594.00	1648.50	1715.00	1860.00
Choroid	1381.00	1565.00	1607.50	1671.00	1744.00
SVC	968.00	1401.00	1486.50	1567.00	1650.00
DVC	1422.00	1714.00	1767.50	1811.00	1918.00
Ret	1535.00	1732.00	1774.50	1834.00	1932.00
Full	1570.00	1882.00	1911.50	1940.00	2027.00
**JD** (mm^2)					
RPCP	43.19	98.80	127.91	152.60	189.36
SVP	144.53	175.27	181.63	186.62	200.56
ICP	168.60	203.92	208.98	213.33	221.70
DCP	182.31	196.28	202.24	205.73	221.33
CC	182.23	190.21	196.70	204.66	221.92
Choroid	164.77	186.79	191.81	199.39	208.08
SVC	115.52	167.16	177.39	187.02	196.87
DVC	169.66	204.53	210.89	216.11	228.88
Ret	183.14	206.66	211.75	218.82	230.51
Full	187.34	224.59	228.08	231.51	241.85
**TVL** (mm)					
RPCP	60.18	97.41	114.65	130.74	149.25
SVP	133.20	147.87	150.55	153.45	160.67
ICP	143.14	160.26	162.37	164.45	170.01
DCP	147.16	155.28	157.96	160.42	166.44
CC	154.35	158.55	161.12	163.73	169.61
Choroid	143.66	153.88	156.86	159.77	163.19
SVC	118.50	143.89	147.76	152.05	158.27
DVC	141.61	160.86	163.26	165.09	170.36
Ret	151.18	160.40	163.14	166.38	170.80
Full	149.63	168.53	170.06	171.67	175.74
**AVL** (mm)					
RPCP	0.13	0.30	0.50	0.75	123.21
SVP	2.20	5.85	7.55	10.35	151.60

ICP	1.54	5.77	7.42	11.91	162.35
DCP	1.60	8.28	9.85	12.54	158.73
CC	6.56	39.50	79.55	82.27	167.17
Choroid	1.94	8.26	13.89	23.04	158.72
SVC	0.89	4.14	6.47	8.79	152.12
DVC	1.37	10.25	19.30	33.57	164.48
Ret	1.81	4.30	5.66	8.79	166.66
Full	1.50	15.26	21.23	34.44	171.60
**TNEP (N)**					
RPCP	395.00	699.00	797.00	945.00	1177.00
SVP	78.00	126.00	145.50	175.00	346.00
ICP	66.00	131.00	155.50	184.00	373.00
DCP	77.00	110.00	126.50	149.00	367.00
CC	26.00	49.00	60.00	70.00	229.00
Choroid	41.00	161.00	191.00	243.00	383.00
SVC	109.00	151.00	180.00	243.00	588.00
DVC	43.00	75.00	90.50	110.00	383.00
Ret	89.00	155.00	188.50	247.00	424.00
Full	70.00	122.00	154.00	187.00	506.00
**L**					
RPCP	0.06	0.10	0.15	0.22	0.43
SVP	0.02	0.03	0.04	0.05	0.06
ICP	0.01	0.02	0.02	0.02	0.06
DCP	0.01	0.03	0.04	0.04	0.06
CC	0.01	0.01	0.01	0.01	0.02
Choroid	0.01	0.01	0.01	0.02	0.04
SVC	0.02	0.04	0.05	0.06	0.09
DVC	0.01	0.02	0.02	0.03	0.06
Ret	0.01	0.02	0.03	0.03	0.05
Full	0.01	0.01	0.01	0.01	0.06
**FAZ area** (mm^2)					
RPCP	0.44	0.81	1.20	1.49	2.89
SVP	0.18	0.39	0.54	0.65	0.83
ICP	0.00	0.13	0.22	0.30	0.43
DCP	0.11	0.40	0.53	0.61	0.90
SVC	0.22	0.45	0.64	0.75	1.04

DVC	0.00	0.17	0.30	0.36	0.53
Ret	0.00	0.17	0.30	0.35	1.03
**VDI** (µm)					
RPCP	25.07	26.57	27.40	28.82	32.69
SVP	32.54	34.65	35.96	36.70	37.82
ICP	30.34	32.85	33.45	34.31	36.10
DCP	32.42	34.39	35.70	36.47	41.00
CC	34.25	39.91	41.61	42.90	44.36
Choroid	31.70	33.73	35.12	35.97	41.94
SVC	29.15	32.22	33.55	34.53	35.72
DVC	31.56	34.77	35.76	36.85	39.43
Ret	29.20	31.09	32.28	33.36	34.85
Full	28.73	31.95	32.81	33.80	36.00
**T**					
RPCP	3.13	5.59	8.20	11.76	1884.00
SVP	26.21	64.28	82.61	116.92	1664.00
ICP	19.53	69.86	86.93	141.00	1920.00
DCP	20.70	95.84	113.90	143.33	1812.00
CC	74.33	418.75	824.75	917.50	1873.00
Choroid	23.82	96.53	160.20	267.86	1932.00
SVC	11.69	48.33	74.00	99.12	1701.00
DVC	17.51	120.13	220.92	386.20	1879.00
Ret	22.43	51.71	67.95	106.63	1953.00
Full	20.75	182.91	260.44	408.60	2084.00

n number of participants, *EA* explant area, *VA* vessels area, *VD* vessels density, *TNJ* total number of junctions, *JD* junctions density, *TVL* total vessel length, *AVL* average vessel length, *TNEP* total number of endpoints, *L* mean lacunarity, *FAZ* foveal avascular zone, *VDI* vessel diameter index, *T* tortuosity, *RPCP* radial peripapillary capillary plexus, *SVP* superficial vascular plexus, *ICP* intermediate vascular plexus, *DCP* deep capillary plexus, *SVC* superficial vascular complex, *DVC* deep vascular complex, *CC* choriocapillaris, *Ret* retina, *Full* chorioretina, *CMT* central macular thickness, *CMV* central macular volume, *FoT* foveolar thickness, *PTT* parafoveal temporal thickness, *PTV* parafoveal temporal volume, *PNT* parafoveal nasal thickness, *PNV* parafoveal nasal volume, *PST* parafoveal superior thickness, *PSV* parafoveal superior volume, *PIT* parafoveal inferior thickness, *PIV* parafoveal inferior volume.

Table S2 Distribution of macular thickness and volume

Variable (measurement unit)	Mean	Median	SD	Minimum	Maximum	Percentiles		
						25th	50th	75th
CMT* (μm)	253.82	253.50	18.81	220.00	306.00	239.75	253.50	265.00
CMV* (μl)	0.20	0.20	0.01	0.17	0.24	0.19	0.20	0.21
FoT (μm)	206.52	206.50	25.36	50.00	266.00	199.00	206.50	215.25
PFT (μm)	324.63	327.00	13.24	285.00	350.00	318.25	327.00	333.00
PFV (μl)	0.51	0.51	0.02	0.45	0.55	0.50	0.51	0.52
PNT (μm)	335.39	335.50	15.33	288.00	371.00	325.00	335.50	345.25
PNV (μl)	0.53	0.53	0.02	0.45	0.58	0.51	0.53	0.54
PST (μm)	338.03	338.50	13.05	300.00	371.00	330.50	338.50	347.25
PSV (μl)	0.53	0.53	0.02	0.47	0.58	0.52	0.53	0.55
PIT (μm)	334.32	335.50	13.67	296.00	360.00	327.00	335.50	343.25
PIV (μl)	0.53	0.53	0.02	0.47	0.57	0.51	0.53	0.54

CMT central macular thickness, *CMV* central macular volume, *FoT* foveolar thickness, *PTT* parafoveal temporal thickness, *PTV* parafoveal temporal volume, *PNT* parafoveal nasal thickness, *PNV* parafoveal nasal volume, *PST* parafoveal superior thickness, *PSV* parafoveal superior volume, *PIT* parafoveal inferior thickness, *PIV* parafoveal inferior volume, *SD* standard deviation.

*Value measured accross diameter of central 1 mm.

Table S3 Post-hoc analysis of the examined variables

Variable	Mean rank	Different (P<0.05) from variable number
**VA (mm^2)**		
(1) CC	9.84	(2) (3) (4) (5) (6) (7) (8) (9) (10)
(2) Choroid	5.47	(1) (3) (4) (5) (7) (8) (9) (10)
(3) DCP	6.73	(1) (2) (4) (6) (7) (8) (9) (10)
(4) DVC	8.60	(1) (2) (3) (5) (6) (7) (8) (9) (10)
(5) Full	6.58	(1) (2) (4) (6) (7) (8) (9) (10)
(6) ICP	5.29	(1) (3) (4) (5) (7) (8) (9)
(7) RPCP	1.00	(1) (2) (3) (4) (5) (6) (8) (9) (10)
(8) Ret	4.31	(1) (2) (3) (4) (5) (6) (7) (9)
(9) SVC	2.39	(1) (2) (3) (4) (5) (6) (7) (8) (10)
(10) SVP	4.81	(1) (2) (3) (4) (5) (7) (9)
**VD (%)**		
(1) CC	9.84	(2) (3) (4) (5) (6) (7) (8) (9) (10)
(2) Choroid	5.47	(1) (3) (4) (5) (7) (8) (9) (10)
(3) DCP	6.73	(1) (2) (4) (6) (7) (8) (9) (10)
(4) DVC	8.60	(1) (2) (3) (5) (6) (7) (8) (9) (10)
(5) Full	6.58	(1) (2) (4) (6) (7) (8) (9) (10)
(6) ICP	5.29	(1) (3) (4) (5) (7) (8) (9)
(7) RPCP	1.00	(1) (2) (3) (4) (5) (6) (8) (9) (10)
(8) Ret	4.31	(1) (2) (3) (4) (5) (6) (7) (9)
(9) SVC	2.39	(1) (2) (3) (4) (5) (6) (7) (8) (10)
(10) SVP	4.81	(1) (2) (3) (4) (5) (7) (9)
**TNJ (N)**		
(1) CC	5.50	(2) (3) (4) (5) (6) (7) (8) (9) (10)
(2) Choroid	4.32	(1) (3) (4) (5) (6) (7) (8) (9) (10)
(3) DCP	5.95	(1) (2) (4) (5) (6) (7) (8) (9) (10)
(4) DVC	7.73	(1) (2) (3) (5) (6) (7) (9) (10)
(5) Full	9.94	(1) (2) (3) (4) (6) (7) (8) (9) (10)
(6) ICP	7.13	(1) (2) (3) (4) (5) (7) (8) (9) (10)
(7) RPCP	1.06	(1) (2) (3) (4) (5) (6) (8) (9) (10)
(8) Ret	7.87	(1) (2) (3) (5) (6) (7) (9) (10)
(9) SVC	2.52	(1) (2) (3) (4) (5) (6) (7) (8) (10)
(10) SVP	2.98	(1) (2) (3) (4) (5) (6) (7) (8)(9)

**JD (mm²)**		
(1) CC	5.50	(2) (3) (4) (5) (6) (7) (8) (9) (10)
(2) Choroid	4.32	(1) (3) (4) (5) (6) (7) (8) (9) (10)
(3) DCP	5.94	(1) (2) (4) (5) (6) (7) (8) (9) (10)
(4) DVC	7.73	(1) (2) (3) (5) (6) (7) (9) (10)
(5) Full	9.94	(1) (2) (3) (4) (6) (7) (8) (9) (10)
(6) ICP	7.13	(1) (2) (3) (4) (5) (7) (8) (9) (10)
(7) RPCP	1.06	(1) (2) (3) (4) (5) (6) (8) (9) (10)
(8) Ret	7.89	(1) (2) (3) (5) (6) (7) (9) (10)
(9) SVC	2.52	(1) (2) (3) (4) (5) (6) (7) (8) (10)
(10) SVP	2.98	(1) (2) (3) (4) (5) (6) (7) (8) (9)
**TVL (mm)**		
(1) CC	6.60	(2) (3) (4) (5) (7) (8) (9) (10)
(2) Choroid	4.82	(1) (4) (5) (6) (7) (8) (9) (10)
(3) DCP	5.06	(1) (4) (5) (6) (7) (8) (9) (10)
(4) DVC	7.52	(1) (2) (3) (5) (6) (7) (9) (10)
(5) Full	9.87	(1) (2) (3) (4) (6) (7) (8) (9) (10)
(6) ICP	7.05	(2) (3) (4) (5) (7) (8) (9) (10)
(7) RPCP	1.02	(1) (2) (3) (4) (5) (6) (8) (9) (10)
(8) Ret	7.63	(1) (2) (3) (5) (6) (7) (9) (10)
(9) SVC	2.27	(1) (2) (3) (4) (5) (6) (7) (8) (10)
(10) SVP	3.16	(1) (2) (3) (4) (5) (6) (7) (8) (9)
**AVL (mm)**		
(1) CC	9.44	(2) (3) (4) (5) (6) (7) (8) (9) (10)
(2) Choroid	6.42	(1) (3) (4) (5) (6) (7) (8) (9) (10)
(3) DCP	5.52	(1) (2) (4) (5) (6) (7) (8) (9) (10)
(4) DVC	7.79	(1) (2) (3) (6) (7) (8) (9) (10)
(5) Full	8.02	(1) (2) (3) (6) (7) (8) (9) (10)
(6) ICP	4.87	(1) (2) (3) (4) (5) (7) (8) (9)
(7) RPCP	1.00	(1) (2) (3) (4) (5) (6) (8) (9) (10)
(8) Ret	3.86	(1) (2) (3) (4) (5) (6) (7)
(9) SVC	3.68	(1) (2) (3) (4) (5) (6) (7) (10)
(10) SVP	4.42	(1) (2) (3) (4) (5) (7) (9)
**TNEP (N)**		
(1) CC	1.37	(2) (3) (4) (5) (6) (7) (8) (9) (10)

(2) Choroid	6.90	(1) (3) (4) (5) (6) (7) (10)
(3) DCP	4.02	(1) (2) (4) (5) (6) (7) (8) (9) (10)
(4) DVC	2.26	(1) (2) (3) (5) (6) (7) (8) (9) (10)
(5) Full	5.28	(1) (2) (3) (4) (7) (8) (9)
(6) ICP	5.67	(1) (2) (3) (4) (7) (8) (9) (10)
(7) RPCP	10.00	(1) (2) (3) (4) (5) (6) (8) (9) (10)
(8) Ret	7.09	(1) (3) (4) (5) (6) (7) (10)
(9) SVC	7.41	(1) (3) (4) (5) (6) (7) (10)
(10) SVP	5.00	(1) (2) (3) (4) (6) (7) (8) (9)
**L**		
(1) CC	1.06	(2) (3) (4) (5) (6) (7) (8) (9) (10)
(2) Choroid	3.11	(1) (3) (4) (5) (6) (7) (8) (9) (10)
(3) DCP	7.31	(1) (2) (4) (5) (6) (7) (8) (9)
(4) DVC	5.11	(1) (2) (3) (5) (6) (7) (9) (10)
(5) Full	2.37	(1) (2) (3) (4) (6) (7) (8) (9) (10)
(6) ICP	4.23	(1) (2) (3) (4) (5) (7) (8) (9) (10)
(7) RPCP	10.00	(1) (2) (3) (4) (5) (6) (8) (9) (10)
(8) Ret	5.42	(1) (2) (3) (5) (6) (7) (9) (10)
(9) SVC	8.81	(1) (2) (3) (4) (5) (6) (7) (8) (10)
(10) SVP	7.58	(1) (2) (4) (5) (6) (7) (8) (9)
**FAZ area (mm^2)**		
(1) DCP	4.63	(2) (3) (4) (5) (6)
(2) DVC	2.35	(1) (3) (4) (6) (7)
(3) ICP	1.17	(1) (2) (4) (5) (6) (7)
(4) RPCP	6.94	(1) (2) (3) (5) (6) (7)
(5) Ret	2.52	(1) (3) (4) (6) (7)
(6) SVC	5.85	(1) (2) (3) (4) (5) (7)
(7) SVP	4.53	(2) (3) (4) (5) (6)
**VDI (µm)**		
(1) CC	9.86	(2) (3) (4) (5) (6) (7) (8) (9) (10)
(2) Choroid	6.18	(1) (3) (4) (5) (6) (7) (8) (9) (10)
(3) DCP	7.34	(1) (2) (5) (6) (7) (8) (9)
(4) DVC	7.76	(1) (2) (5) (6) (7) (8) (9)
(5) Full	3.48	(1) (2) (3) (4) (6) (7) (9) (10)
(6) ICP	4.61	(1) (2) (3) (4) (5) (7) (8) (10)
(7) RPCP	1.05	(1) (2) (3) (4) (5) (6) (8) (9) (10)

(8) Ret	3.00	(1) (2) (3) (4) (6) (7) (9) (10)
(9) SVC	4.13	(1) (2) (3) (4) (5) (7) (8) (10)
(10) SVP	7.58	(1) (2) (5) (6) (7) (8) (9)
**T**		
(1) CC	9.34	(2) (3) (4) (5) (6) (7) (8) (9) (10)
(2) Choroid	6.53	(1) (3) (4) (5) (6) (7) (8) (9) (10)
(3) DCP	5.55	(1) (2) (4) (5) (7) (8) (9) (10)
(4) DVC	7.76	(1) (2) (3) (6) (7) (8) (9) (10)
(5) Full	8.08	(1) (2) (3) (6) (7) (8) (9) (10)
(6) ICP	4.94	(1) (2) (4) (5) (7) (8) (9) (10)
(7) RPCP	1.08	(1) (2) (3) (4) (5) (6) (8) (9) (10)
(8) Ret	3.98	(1) (2) (3) (4) (5) (6) (7)
(9) SVC	3.60	(1) (2) (3) (4) (5) (6) (7)
(10) SVP	4.15	(1) (2) (3) (4) (5) (6) (7)

EA explant area, *VA* vessels area, *VD* vessels density, *TNJ* total number of junctions, *JD* junctions density, *TVL* total vessel length, *AVL* average vessel length, *TNEP* total number of endpoints, *L* mean lacunarity, *FAZ* foveal avascular zone, *VDI* vessel diameter index, *T* tortuosity, *RPCP* radial peripapillary capillary plexus, *SVP* superficial vascular plexus, *ICP* intermediate vascular plexus, *DCP* deep capillary plexus, *SVC* superficial vascular complex, *DVC* deep vascular complex, *CC* choriocapillaris, *Ret* retina, *Full* chorioretina.

*Statistical significance was measured using Conover post-hoc test ($P < 0.05$).

Table S4 Globe biometry normative values in 4-year-old Caucasian children (*n* = 62)

Component (measurement unit)	Mean	SD	Minimum	Maximum
AL (mm)	22.17	0.53	20.99	23.59
ACD (mm)	3.42	0.27	2.74	4.12
LT (mm)	3.69	0.19	3.38	4.21
K1 (D)	42.03	1.17	39.48	45.22
K2 (D)	43.85	1.29	39.72	46.46
SE (D)	43.44	1.21	39.60	45.65
CCT (μm)	539.21	30.15	461.00	604.00
WTW (mm)	12.29	0.37	11.40	13.20
IOL (D)*	25.76	1.25	22.50	30.00

AL axial length, *ACD* anterior chamber depth, *LT* lens thickness, *K1* flat corneal meridian, *K2* steep corneal meridian, *SE* spherical equivalent, *CCT* central corneal thickness, *WTW* white-to-white, *IOL* intraocular lens, *SD* standard deviation, *n* number of participants.

*Acrysoft IQ monofocal SN60WF (Alcon Laboratories Inc, Fort Worth, Texas, USA), A-constant 118.7 using SRK-T formula.
